# Legal, deontological, and ethical basis of the physician–minor patient care relationship

**DOI:** 10.3389/fmed.2023.1186414

**Published:** 2023-05-25

**Authors:** Clara Cestonaro, Anna Aprile

**Affiliations:** Legal Medicine, Department of Cardiac, Thoracic, Vascular Sciences and Public Health, University of Padova, Padua, Italy

**Keywords:** healthcare, confidentiality, protection, law, minors

## 1. Introduction

The relationship between healthcare professionals and minors is a complex issue that refers to awareness and decision-making autonomy and on which legal, deontological, and ethical aspects intersect. The physician–patient relationship has long been characterized by an asymmetrical interaction, where the physician took a dominant position over the patient, and only recent decades have given way to a new model in which patients assume a more active and autonomous role with increased mutual participation in the relationship (1). Physicians are required to inform patients about their medical condition, refer them to an adequate specialist if needed, and obtain their consent for medical and surgical treatment. Whereas in adults the ability to understand the information provided by the physician is presumed, as is the ability to make free and informed decisions, unanimous agreement on the age at which a minor can be deemed competent for decision-making does not exist and the age at which minors can consent to medical treatments also without parental consent varies among countries (2). Despite being the same age, minors' maturity may differ, and their decision-making competence is also affected by context (3). In fact, even the evaluation of unaccompanied minors' age using psychological tests and non-medical methods is affected by environmental and cultural factors (so that an assessment including forensic expertise is suggested in these cases) (4). Historically, the right to make decisions for minors was vested in their parents or guardians; however, over the past century, a recognition of minors' role in the healthcare context emerged, including in matters of sexual and reproductive health. Confidentiality is a cornerstone of healthcare, such as autonomy, privacy, and consent; nevertheless, when addressing minors' care, clinicians may not be clear whether this principle also applies to them (5–7). The limits within which the doctor–minor patient relationship currently moves may cause uncertainty in doctors, minors, and parents, creating an impact on patient care. This study purposes to review what legal, deontological, and ethical references currently characterize the doctor–minor patient relationship in the Italian context, with particular reference to the area of sexual and reproductive health.

## 2. Legal, deontological, and ethical references

### 2.1. Legal references

The Italian legal system contains multiple references to minors' autonomy in areas that impact the relationship with a healthcare professional, which are contained in both the penal code and specific laws. A reference of primary importance consists in Law n. 194 of 1978 (8), which explicitly deals with minors in relation to contraceptive administration, at Article 2, and to the termination of pregnancy, at Article 12. Both these services are accessible because of this law by the minor, whose lower age limit is not specified. According to the 1978 Law, the administration on medical prescription in health facilities of the ‘means necessary to achieve the purposes freely chosen for responsible procreation' was granted to minors. The sale of emergency contraception to minors was allowed even in the absence of a medical prescription as a result of a resolution of the Italian Agency of the Drug (*Agenzia Italiana del Farmaco*) of 2020 (9), which was later confirmed by jurisprudence (10). In accordance with the provisions of Law 194/78, whereas in cases of termination of pregnancy requested within the first 90 days of pregnancy, the assent of the adult who is legally responsible for the girl is required, after this limit, the procedures for accessing the intervention are the same as in the adult woman, regardless of the abovementioned assent. Moreover, within the first 90 days of pregnancy, if serious reasons make it impossible or unadvisable to consult the minor's legal guardians or if they refuse the assent or express dissenting opinions, the request is nevertheless forwarded by the doctor to a judge who after also listening to the girl may authorize her to decide the intervention. Assent is also not needed when the intervention is urgent due to the presence of a danger to the minor's health. Whatever the time of pregnancy, the basic prerequisite for performing the termination of pregnancy in the minor is her personal request.

Another key reference on the subject of minors' autonomy with health implications is Article 609-quater of the Italian penal code (11), concerning the crime of ‘sexual acts with minor', which since 2019 has become an ‘*ex officio*' prosecutable crime (12), that is prosecutable even without a complaint by the offended person. According to Article 609-quarter, the existence of the crime depends on the occurrence of sexual acts with a person under 14 years of age or, when the offender is a person with whom the minor has a specific relationship (e.g., authority or guardian), with a person under 16 years of age. An exception is made for cases in which the sexual act is performed by a minor with another minor who reached 13 years of age and between whom the age difference is no more than 4 years. This crime also exists when the sexual acts are performed with a minor who has turned 14 years old by abusing trust/authority/influence, or with a minor who has turned 16 years old if the person with whom the minor has a specific relationship abuses the powers of his/her position.

Law n. 219 of 2017 (13) represents the latest reference addressing the issue of a minor patient–doctor relationship, with particular reference to consent. This Law specifically states, in Article 3, the right of the minor to have his/her capacity for understanding and decision-making enhanced, and the need to receive health information appropriately to be enabled to express own will. Consent to health treatments shall be given or refused by whoever is legally responsible for the minor considering the minor's will and aiming at protecting his/her physical and psychical health, respecting his/her dignity.

### 2.2. Deontological and ethical references

The physicians' professional oath includes a commitment to pursue with the person being cared for a caring relationship based on trust and respect for each person's values and rights and on information, prior to the consent, that is understandable and complete, as well as a commitment to protect the confidentiality of everything confided. The physician code of deontology stipulates that the relationship between the physician and the patient should be based on freedom of choice and the identification of respective autonomies and responsibilities. The minor is included among so-called fragile subjects who should be protected by the physician, on whom also falls the responsibility to report situations of abuse (14).

From the ethical point of view, the principle of respect for autonomy involves “the recognition of the right to make choices and take actions based on personal values and beliefs.” The physician who adheres to this principle communicates information, ascertains its understanding, and encourages adequate decision-making. The physician who respects patient's autonomy moreover acts to provide him/her with the means necessary to overcome a feeling of dependence and to control the situation as best as possible (15).

## 3. Discussion

Minor care usually implies a triad consisting of clinician, minor, and parents (16); as the minor grows up, however, a direct relationship with the clinician becomes established, and confidential care issues emerge. Confidentiality indicates the ‘protection of privileged and private information shared during a healthcare encounter and in medical records that document the encounter' and relates to the principle of respect for autonomy (17). The importance of confidential care for adolescents has been highlighted by the American College of Obstetricians and Gynecologists (ACOG) as it is considered to encourage access to care and increase discussions of issues that impact the wellbeing of the minor (18). Concerns related to confidentiality have multiple repercussions in seeking healthcare; they may impact the decision to use emergency contraception, can lead to delays in abortion, and may represent a barrier to discussions related to intimate partner violence (19). Because of the fear of a lack of confidentiality, some adolescents may be discouraged from seeking health counseling, especially when dealing with sensitive issues such as contraception, sexual activities, depression, or illicit substance use (20). The American Academy of Family Physicians (AAFP) therefore recommends the delivery of confidential health services ‘in situations involving reproductive health, sexuality, gender identity and expression, substance use, and mental health to consenting adolescents' (21). Confidential care for adolescents may achieve more success if healthcare professionals build trust both with parents and the minors to simplify the parental acceptance of the confidential care itself (22). Minors' willingness to be open may be improved by the doctor's explanation concerning the confidentiality of the discussion, and when confidentiality cannot be guaranteed, it would be the physician's responsibility to explain the reasons (20). Adolescent patients in particular should be made aware that some circumstances limit the guarantee of confidentiality.

In the Italian context, respect for confidentiality toward the minor in matters of reproductive health is peculiar not only on the basis of ethical and deontological provisions but also of specific laws regarding contraception, termination of pregnancy, and sexual acts. Identification of legal and ethical support for confidentiality does not, however, exempt the physician from taking actions to protect the minor in cases where his/her request reveals grounds for concern, for example, referring to the relationship with an abusive adult.

Situations that give rise to suspicion of a crime represent reasons why the physician may not respect confidentiality. According to the Law, the physician providing care in cases having characteristics attributable to an *ex officio* prosecutable crime must report it to the judicial authority (23). *Ex officio* prosecutable crimes are those whose seriousness is such that they require activation regardless of the complaint of the offended person, and they include not only murders but also some types of sexual violence (such as against people under 18 years of age), sexual acts with minors, and maltreatment. Knowledge of which circumstances constitute one of these crimes is fundamental for the clinician to know when respect for confidentiality should be exceeded and a report is needed. The establishment by the penal code of an age limit below which the performance of a sexual act (even without violence) constitutes an offense is read as the identification of an age beyond which the minor is deemed to have sufficient awareness to freely engage in sexual relations. This age is deemed to be greater in cases where there is a special relationship between the two parties, for example, a relationship of cohabitation, education, and care. Exceeding this age, however, does not preclude the existence of situations for which confidentiality must be overcome, and clinicians should not only assess the age of the adolescents and their partner but also perform an individual evaluation, considering the consent of the minor and the existence of any signs attributable to violence (24). Clinicians providing care to an adolescent with whom a caring relationship is established must be able to detect those signs, including those other than somatic lesions, that might underlie a condition that necessitates protection of the minor and must be clear in relation to what situations require reporting to a judicial authority.

## 4. Conclusion

Confidentiality is a fundamental element of the doctor–patient relationship, even when the patient is an adolescent, and positively impacts minors' trust and access to care. Legal, deontological, and ethical bases for confidentiality, however, do not eliminate the fact that acting responsibly may direct the clinician to overcome the confidentiality toward the minor in cases where his/her request reveals grounds for concern, for example, referring to a relationship with an abusive adult.

## Author contributions

AA and CC: conceptualization. CC: writing—draft preparation and editing. AA: supervision. All authors have read and agreed to the published version of the manuscript.
